# Diseases of the Antrum Maxillare

**Published:** 1836

**Authors:** S. P. Hullihen

**Affiliations:** Dentist, Wheeling, Va.


### Article IV.—Diseases of the Antrum Maxillare.

*Observations on Abscess, Purulent Secretion, and Fungous Tumors of the Antrum Maxillare.* By S. P. Hullihen, Dentist, Wheeling, Va.

The mucus membrane lining the antrum maxillare is frequently the seat of disease produced by some local exciting cause. The most common causes of diseases in this cavity, are the fangs of the molares, which frequently penetrate the antrum, and coming in contact with its membrane, produce what has been sometimes very properly termed "abscess of the antrum maxillare." Mr. Bell, in speaking of this disease, considers it to be nothing more than an altered secretion of the membranes, and says, "The term abscess as applied to the disease in question has given rise to very mistaken notions of its nature, and not less erroneous principles of treatment. That a mucus membrane covering in a thin layer the whole internal surface of such a cavity, should become the seat of all the consecutive steps of true abscess, is a statement bearing on the face of it an obvious absurdity." (Bell on the Teeth. p. 253.)

This notion of Mr. Bell's is surely as erroneous as any that has ever been advanced upon the subject. A mere glance at the origin of the disease will satisfy the most superficial observer that it is in the strictest sense of the word, abscess, and must be considered as perfectly identical with alveolar abscess, both proceeding from the same cause, and the progression and development of both being the same. In offering this portion of my remarks to the medical public, I am well aware that I tread on ground untrodden before but hope my success in practice, and the facts I shall adduce, will be a sufficient apology for my heterodoxy in doctrine. The primary cause of abscess in the antrum as well as of the alveolar process, consists in the suppuration of the nerves and vessels situated within the internal cavity of a tooth. This suppuration is the result of inflammation, induced either by a blow upon the tooth so as to rupture its vessels; the fracture of a tooth; the pressure of a plug upon the nerve; the existence of a cavity produced by caries and extending very near the nerve, rendering it liable to irritation from the lodgement of food; or extreme degrees of heat or cold. When the pus thus formed escapes through an opening in the crown of the tooth, the bad effects which would otherwise have supervened are obviated. But if the pus be so confined as to prevent its escape through the crown, it immediately commences to ooze out at the extremity of the fang, where the nerves and vessels entered, and coming in contact with the periosteum it separates it from the fang, and a sac is formed precisely at the foramen. The sac now enlarges by an accumulation of pus secreted from the internal membrane of the tooth, until it bursts. If the fangs of such a tooth penetrate the antrum, the membrane of this cavity being always continuous over the fangs, the pus is effused between the membrane and the bone, thus forming a *true abscess* in the antrum. The pus now accumulates by secretion from the surrounding parts, until the distended membrane bursts, and the pus escapes from the antrum through the duct into the nose. On the other hand, if the fangs of a tooth containing pus, terminate properly in the cells of the alveoli, the distension of the sac producing great pressure upon the surrounding parts, causes the external plate (being the thinner) to be absorbed. Thus as the sac enlarges and bursts, the pus is effused between the alveolus and its periosteum, thereby forming what has been generally termed, "Alveolar abscess." After the contents of an abscess are first discharged through the gums there continues to be a fistulous opening through which a small quantity of pus is discharged as long as the diseased fang is suffered to remain. This accounts for the long continued discharge from an abscess in the antrum. The internal cavity of a tooth being lined by a highly vascular membrane, secretes very rapidly, and thus a small quantity of pus is formed and discharged into the antrum for sometime after the bursting of the abscess. This deposition of pus into the antrum is a constant source of irritation to its membrane, and excites in it a diseased action. The discharge from the tooth continues until its internal membrane is entirely destroyed, and then commences the decomposition of the point of the offending fang, and thus the discharge of irritating matter is further continued. In this manner a constant discharge from the antrum into the nose is continued for a number of years, until the functions of the membrane are completely destroyed. Then commences an ulceration of the membrane, from which small fungous granulations arise, which in time produce that very alarming disease called, "Fungous tumors of the Antrum Maxillare." An abscess of the antrum, may also be occasioned by mechanical injuries; such as fracture of the malar bone, a blow on the under jaw driving a molar tooth upwards and causing its fangs to enter the antrum. Such cases however, it must be acknowledged are very rare. But from whatever cause an abscess may form, if its cure be neglected it will generally terminate in fungous tumors.

"Inflammation of this membranous lining of the antrum," says Cooper, speaking of abscess in this cavity, "sometimes produces an extraordinary secretion of mucus within it." (Vid Dict. p. 151.) "This disease," says Boyer, describing a collection of mucus within the antrum and terming it abscess; "is sometimes ascribed to a blow upon the cheek, to caries of the teeth, or the projection of one of their fangs into the antrum, but in general the cases take place unprecedented by any of these causes, and without there being the least ground for suspecting what has given rise to the disease." (Traite des maladis, Chir. tom. 6, p. 131.) Jourdain, Deschamps, Fox, Default, Abernethy, Gibson and others in noticing this disease all coincide with its above described causes, and all agree in terming it abscess.

Thus it appears that a collection of pus in the antrum maxillare, proceeding from any and every cause has been universally termed abscess by all writers on the subject except Mr. Bell, who, as I have shown committed as great an error as the one he strove to correct.

I will now endeavor to show that there is another collection of pus frequently occuring in this cavity, proceeding from a different cause, and producing very different effects, which it is as erroneous to term abscess, as to deny, like Mr. Bell, the existence of one. This disease I purpose to call, "*Purulent secretion of the antrum maxillares*," a name which expresses its true nature. An abscess of the antrum commences as before described, in suppuration between the bone and its lining membrane; purulent secretion is a disease of the antrum brought on by the closure of its nasal duct, the inflammation produced by the confined mucus promoting only an altered secretion of its membrane. The primary cause of purulent secretion, as I have before remarked, consists in the closure of the nasal duct of the antrum. It must here be remembered that any cause producing excessive inflammation in the rear of the lining membrane of this cavity, such as a blow on the cheek or the projection of a diseased fang, invariably terminates in abscess. Furthermore, inflammation, so slight as not to form an abscess, and proceeding from the same causes that give origin to one, would not be at all likely to thicken the membrane so much as to close the duct, and the effect of such inflammation would only increase the quantity of the mucus, and that too without materially altering its character. On the other hand, an inflammation in the lining membrane of the nose, brought on by colds, or otherwise, would doubtless close the duct before the inflammation could extend into the antrum and cause an altered secretion of its membrane. It is, therefore, evident that purulent secretion is a disease originating in an inflammation and closure of the ductus ad nasum.

Purulent secretion takes place from a variety of causes, and is dependent on inflammation or other disease of the nasal fossæ. A blow on the nose, a wound in the nostril, polypi of the nose, severe colds and ulcerations of the pituitary membrane, are the most common causes of closure of the duct. When the duct first closes, no pain whatever is experienced in the antrum, and indeed not until it becomes *filled* with mucus: then the following are the most usual symptoms—a slight uneasiness in the antrum, with a sensation of great weight or pressure, after which a dull deep seated pain supervenes, which is followed by an acute pain darting into the ear, over the eye, and in the direction of the frontal sinus, accompained by a greater or less degree of tumefaction of the cheek. After this period of the disease, the walls of the antrum subjected to the long and increasing pressure of the accumulated secretion, give way and the purulent matter escapes through the opening. This generally occurs from one to three months after the closure of the duct. Abscess of the antrum is accompanied by very different symptoms. The patient first imagines he has the toothache, from a dull throbbing pain over a tooth, which he will most likely point out as being sore. This may continue for a day or two, then a swelling of the cheek commences, which usually increases until the membrane of the antrum breaks, and the pus passes through the duct into the nose. This discharge of the abscess usually occurs in three or four days after its first commencement. Abscess of the antrum may be easily distinguished from *purulent secretion*, independent of the difference in the symptoms of the two diseases. When there is an abscess of the antrum, its nasal duct, I have great reason to believe, is never closed in consequence of the disease; whereas in purulent secretion the duct is always closed. The first discharge of an abscess is usually well digested pus of a very slight fœtor, that of purulent secretion is of a dark color, a slimy consistency, and a fœtor of the rankest kind. By extracting a tooth penetrating the antrum, the contents of the abscess always follow; a discharge of purulent secretion, never does occur from the extraction of any tooth. Abscess of the antrum gives little or no pain after its first formation, although there may be a discharge from it for a number of years, producing but little inconvenience to the patient. Purulent secretion is a disease making frightful progress in a few months, bursting the cavity of the antrum and producing caries of its bony parietes. Having now shown that there are two distinct diseases which cause an accumulation of matter in the antrum, I will dismiss this part of my subject, without farther comment, as it is not my intention to prescribe a course necessary to effect their cure. The preceding observations upon their causes will naturally suggest a proper course of treatment.

I now purpose to offer a few observations on fungus tumors of the antrum, a disease which arises as before stated from ulcerations of the lining membrane of the antrum produced by a long continued discharge from an abscess. This ulceration generally occurs in patches over the surface of the membrane, and in time occasions a lesion of its vessels. If the discharge from the antrum be examined at this stage of the disease, small coagulations of a bright red color may be discovered intermixed with pus of a thick consistency. Soon after this occurs, small fungosities sprout up from the ulcers, which may exist for months without any considerable increase. This depends much upon the patient's general health. But when once these morbid affections become confined, should the slightest irritation occur, their mushroon like growth is truly astonishing. When the antrum becomes filled with a fungus growth, and the cheek begins to enlarge, the symptoms very much resemble those of purulent secretion. But it may always be distinguished from that disease, by a discharge through the nose, and by a fœtor peculiar to fungus affections. As soon as the existence of a fungus tumor in the antrum is discovered, arrangements should be made to exterminate it immediately, and all care should be taken to remove it entirely by the first operation; for should you fail in the first attempt, you will experience greater difficulty in the second. There are cases, however, where the fungus appears again and again, in defiance of the greatest care the operator may bestow upon its removal. But among several which I have had under my care, I have met with but one of this obstinate character. This case presented several rather remarkable circumstances in the progress of its cure, which induces me to detail it at length.

May, 1836. Jackson D. Shipley a highly respectable gentleman of this city, called to consult me, in relation to a diseased antrum which originated fourteen years before, from the injudicious extraction of a tooth. For the last three months, he had experienced all the symptoms which usually attend the first stages of a fungus growth. This, in connexion with the fœtor of his breath, induced me unhesitatingly to pronounce it a fungus affection of the antrum, and I urged an operation accordingly; but his previous engagements were of such a nature as not to admit of it at this time.

June 3d. Mr. Shipley called again with his attending physician, in order to have the operation performed on his antrum. After all the necessary arrangements were made I extracted the second bicuspidati, which had the appearance of total necrosis. I then perforated the floor of the antrum through the alveolar cell; the trochar making an opening which would admit a common buck-shot. Upon introducing a probe through this opening, I discovered that the tumor had nearly filled the cavity, but as soon as I touched it the patient fainted, and then passed into a severe convulsion. It was now deemed inexpedient to continue the operation at this time, as the slightest touch on the tumor gave the most exquisite pain. The patient was therefore put under proper medical treatment by his physician, during which time a decoction of poppy leaves was injected twice a day.

June 13th. Patient's health much better; upon introducing a probe into the antrum, not the slightest pain was experienced. The operation was now continued. The patient being seated on an operating chair, his head reclining against the back, I removed the gums with a scalpel, as far as was necessary, and then sawed out the alveoli and floor of the antrum together. This was done by means of a small saw made expressly for the purpose, about the size of a common penknife blade, which was introduced into the opening first made, from which I sawed along the floor and inner plates of the alveoli, to the last molaris, where an opening had been made at the commencement of the operation. From this opening I sawed outwards and then inwards to the cuts made with the saw from the first opening. By this means I was enabled to remove the detatched bone with my fingers.* The cavity of the antrum being now exposed from the second bicuspis to the last molaris, I removed the tumors by means of suitable scalpels made expressly for that purpose. The hæmorrhags was profuse, but was soon checked by the application of the actual cautery which I used unsparingly on such parts as had the least appearance of fungus. Each time after the application of the cautery, cold water was immediately injected by an assistant.

17th. The fungus re-appeared in several places. Removed by knife, and actual cautery applied.

19th. Fungus growing rapidly, removed, and all suspicious places touched with caustic potash. Health bad.

21st. Fungus removed. The application of caustic potassæ continued.

25th. Actual cautery applied to the tumors which appeared again in several places.

28th. The fungus appears to be somewhat checked—cautery applied.

July 2d. Patient's health bad. Fungus growing rapidly, touched with caustic potassæ.

4th. Antrum so filled with fungus as to occasion much pain. The opening was enlarged, the tumor removed, and a free application of the actual cautery made to the bleeding surface.

7th. Fungus appeared in several places, actual cautery reapplied.

9th. Fungus sprouting up in the upper part of the antrum, touched with actual cautery.

---

*I believe my method of opening an antrum, preparatory to the removal of a tumor, is entirely new, and has many advantages over the common practice as laid down by Dessault and others, and carried out by Gibson. I was not a little surprised on finding this talented surgeon recommending his readers to follow "the fearless Dessault," who always operated with a chisel and mallet. He says, "as soon therefore, as the nature of the tumor is ascertained, the surgeon should not only determine to remove it, but resolve to set no limits to the sacrifices it may be necessary to make. With this view he must provide himself with several curved and angular scalpels of unusual strength and thickness, two or three cauterizing irons, a key for pulling teeth, chisels, gouges and mallets, etc. * * * * * * * * "His next object should be to remove the molar teeth and their alveolar processes, corresponding with the floor of the antrum. This may be done by the tooth key, or *by two or three strokes of a chisel und mallet.*" (Gibson's surgery, vol. 2, p. 16.) A surgeon who would recommend the employment of such tools in an operation like this, could surely have no objections to trepanning with an auger or amputating with a broad-axe.

12th. The fungus is appearing in every part of the antrum, and the patient's health is so bad as not to admit of its removal.

14th. Patient no better, had a severe convulsion.

15th. Patient somewhat better.

16th. Much better.

17th. Patient's health continues to improve, antrum filled with fungus.

18th. The tumors have increased so much as to occasion considerable pain. Patients health continues to improve.

19th. The tumor was again removed, and I took the greatest care not only to remove every portion of the fungus by the application of actual cautery as usual, *but also to sear over the whole surface of the lining membrane.**

20th. The cheek much swollen, health bad.

25th. No appearance of fungus, suppuration profuse.

29th. Several rather suspicious looking places touched with caustic. This was the last time the fungus made its appearance.

The antrum was now injected twice a day with soap and water, which was occasionally changed to tincture of myrrh, decoction of nut galls, etc. Under this treatment the patient improved rapidly, and in the course of four weeks, the whole cavity of the antrum was completely lined with a new membrane. The patient is now enjoying good health, and has been able long since to attend to his business.

---

* Since writing the above, I have opened another antrum where the tumor appeared to be of the same obstinate character. After removing it effectually, the second time, I at once seared over the whole surface of the membrane. The tumor did not again appear and the patient is now almost well.

September, 1836.

